# Restoration of abnormal sleep EEG power in patients with insomnia disorder after 1Hz rTMS over left DLPFC

**DOI:** 10.3389/fpsyt.2024.1431837

**Published:** 2024-09-10

**Authors:** Xumeng Zhao, Jiayi Liu, Ziqiang Shao, Xiaoyang Liu, Zhen Wang, Kai Yuan, Bingqian Zhang, Yan Li, Xiaona Sheng, Yifei Zhu, Yansu Guo

**Affiliations:** ^1^ Department of Psychosomatic Medicine, The Second Hospital of Hebei Medical University, Shijiazhuang, China; ^2^ School of Life Science and Technology, Xidian University, Xi’an, Shanxi, China; ^3^ Department of Neurology, The Second Hospital of Hebei Medical University, Shijiazhuang, Hebei, China

**Keywords:** insomnia disorder, polysomnography, repetitive transcranial magnetic stimulation, relative power, stage 2 NREM sleep

## Abstract

**Introduction:**

Hyperarousal has been a significant pathophysiological theory related to insomnia disorder (ID), characterized by excessive cortical activation and abnormal electroencephalogram (EEG) power during daytime or sleep. However, there is currently insufficient attention to the EEG power during rapid eye movement (REM) sleep and different stages of non-rapid eye movement (NREM) sleep. Additionally, whether the abnormal sleep EEG power in ID patients can be restored by repetitive transcranial magnetic stimulation (rTMS) remains unclear.

**Methods>:**

Data of 26 ID patients and 26 healthy controls (HCs) were included in the current observational study. The comparisons of relative power between patients and HCs at baseline in each band of each sleep stage and the changes in patients before and after rTMS treatment were performed. The correlations between relative power and behavioral measures of the patients were also investigated.

**Results:**

Abnormalities in sleep EEG relative power in the delta, beta and gamma bands of the patients were observed in NREM2, NREM3 and REM sleep. Correlations were identified between relative power and behavioral measures in ID group, primarily encompassing sleep efficiency, sleep onset latency and depression scores. Post-treatment improvements in relative power of the delta and beta band were observed in NREM2 sleep.

**Discussion:**

The relative power of sleep EEG exhibited a significant correlation with sleep measures in ID patients, and demonstrated notable differences from HCs across the delta, beta, and gamma frequency bands. Furthermore, our findings suggest that rTMS treatment may partially ameliorate relative power abnormalities in patients with ID.

## 1 Introduction

Insomnia disorder (ID) is marked by difficulties in initiating and maintaining sleep, and experiencing early morning awakenings (1). It is currently the most prevalent sleep disorder and a widespread health concern, affecting individuals with an overall prevalence ranging from 4% to 20% (2). The ramifications of insomnia extend beyond mere sleep disturbances, contributing to a heightened risk of mental illness, diminishing daytime function and performances, impacting the quality of life (3).

Hyperarousal theory is one of the major proposed pathophysiology of ID, involving the interaction of psychological and physiological factors expressed in terms of somatic, cognitive and cortical activation, with evidence from multiple areas including neuroimaging, neuroendocrinology and electrophysiology (4, 5). Several studies utilizing spectral analysis have revealed alterations in electroencephalogram (EEG) power observed both during the daytime and night, constituting significant pathological manifestations in individuals with ID, although variations in specific findings across these studies existed (6–9). In particular, the beta and gamma band of cortical electrophysiological signals are assumed to be key features of cognitive activities and sensory information processing, and a rise in EEG power within the beta frequency range can be construed as indicative of cortical hyperarousal (4, 10, 11). Recently, a meta-analysis study provided an objective and statistically based assessment of EEG power spectrum in ID patients, suggesting that insomnia is associated not only with increased activity in beta and gamma bands but also with a spreading of this increase to broader frequency bands (12). Furthermore, the brain oscillations across different frequency bands in distinct sleep stages are associated with diverse physiological significance and processes (13). However, current studies have shown limited focus on EEG power during REM sleep, and a corresponding analysis of distinct stages of NREM sleep has been lacking.

Given the widespread abnormal alterations in EEG power across the scalp in patients with ID, developing protocols with the aim of reversing these patterns may be the key to effectively treating insomnia. The direct modulation of spontaneous electrical activity in the cerebral cortex through transcranial magnetic stimulation (TMS) has gained growing recognition in the realms of clinical psychiatry and neurological disorders (14, 15). It has emerged as a promising approach for addressing ID. Recent studies indicated that repetitive TMS (rTMS) can not only effectively alleviate subjective insomnia symptoms and normalize sleep architecture (16), but also has ability to rewire the disrupted functional connections in patients with ID (17). Low-frequency rTMS is hypothesized to inhibit cortical excitability, yielding therapeutic effects in patients with ID. However, the impact of daytime rTMS treatment on abnormal EEG power during sleep in patients with ID remains unclear. Additionally, the sleep stages at which these abnormalities and potential recovery occurred still require further study.

**Figure 1:**
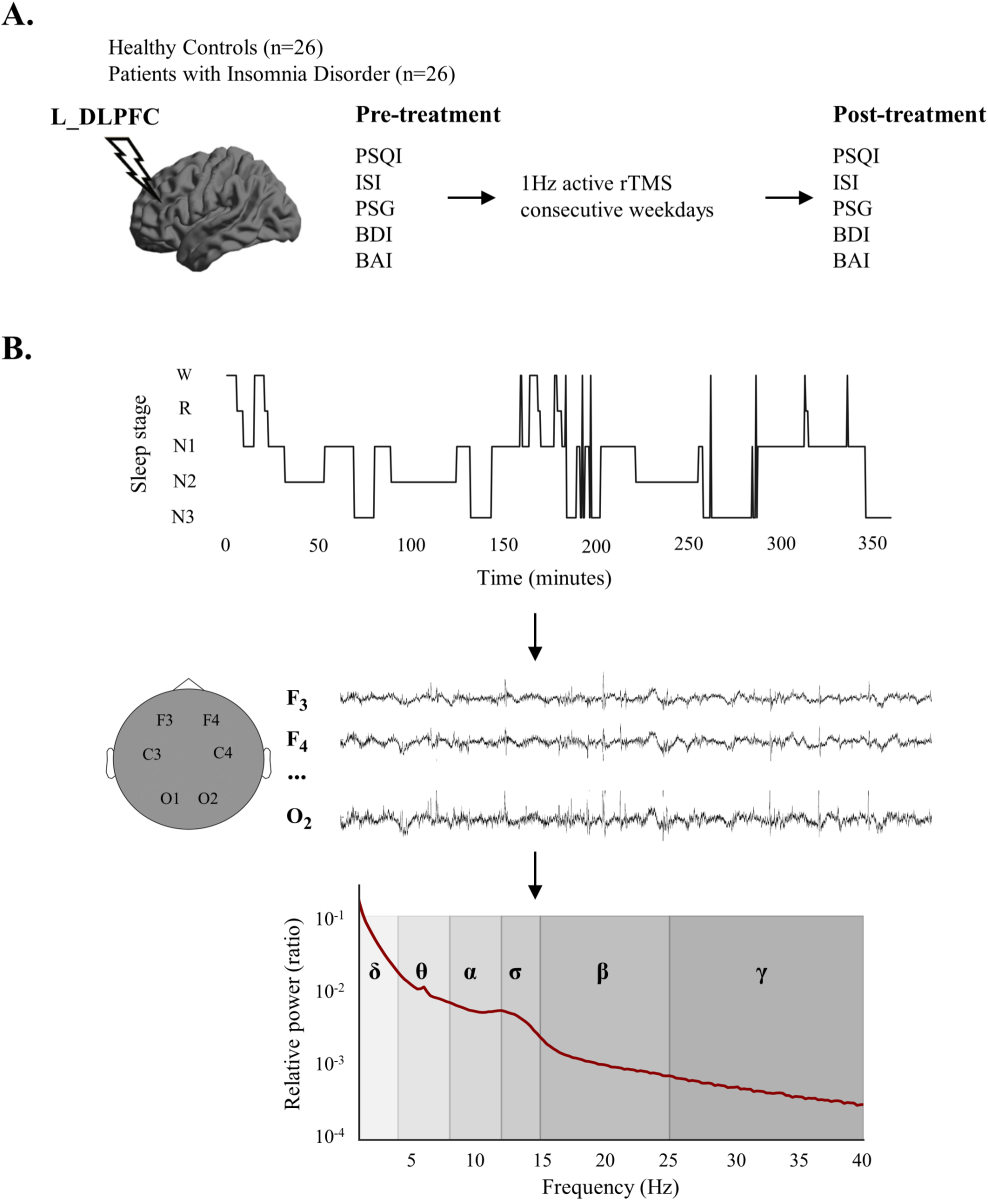
Experimental design and calculation process of sleep electroencephalogram (EEG) relative power. **(A)** Experimental design. **(B)** The relative power calculation process. After sleep stage classification, sleep EEG signals for each sleep stage were obtained at each electrode, followed by the calculation of relative power in various frequency bands. EEG, electroencephalogram; L_DLPFC, left dorsolateral prefrontal cortex; PSQI, Pittsburgh Sleep Quality Index; ISI, Insomnia Severity Index; PSG, polysomnography; BDI, Beck Depression Inventory; BAI, Beck Anxiety Inventory; rTMS, repetitive transcranial magnetic stimulation; W, wake; R, Rapid Eye Movement Sleep; N1, Non-rapid Eye Movement Sleep Stage 1; N2, Non-rapid Eye Movement Sleep Stage 2; N3, Non-Rapid Eye Movement Sleep Stage 3.

In order to further elucidate the effect of rTMS on the cortical electrophysiology of patients with ID, we hypothesized that the patients have abnormalities in a wide range of EEG power spectrum at different stages during sleep, and the abnormal EEG spectral patterns would be partially normalized after the rTMS treatment. Therefore, we conducted an observational study. Patients with ID who received 1Hz rTMS of the left dorsolateral prefrontal cortex (DLPFC) were investigated. Overnight polysomnography (PSG) data and clinical information of the patients before and after treatment were both collected, and spectral analysis of sleep EEG was conducted. The experimental design and sleep EEG relative power calculation process were shown in **Figure 1**.

## 2 Materials and methods

The study was conducted according to the guidelines of the Declaration of Helsinki, and approved by the ethics committee of the Second Hospital of Hebei Medical University (Approval Letter No.2024-R123 and date of approval: 2024.2.23). Informed consent was obtained from all subjects involved in the study.

### 2.1 Participants

26 patients with ID and 26 healthy controls (HCs) matched for age, sex and education were included in the current study. All the participants were either patients attending the hospital or individuals undergoing routine health check-ups.

The inclusion criteria for patients were as follows: (1) age 18–65 years, right-handed; (2) meeting the diagnostic criteria for ID in the Diagnostic and Statistical Manual of Mental Disorders, Fifth Edition (DSM-V); (3) the Pittsburgh Sleep Quality Index (PSQI) > 5; (4) the Insomnia Severity Index (ISI) > 8; (5) did not receive any other treatment (such as cognitive behavioral therapy, medication, etc.) in the two weeks prior to and during the rTMS intervention. The inclusion criteria for healthy subjects were as follows: (1) aged 18–65 years, right-handed; (2) PSQI < 5; (3) ISI < 8; (4) no symptoms or history of psychiatric disorders or sleep disorders and not taking any psychotropic medications or hypnotics during their lifetime. Patients and healthy controls were excluded if they met any of the following criteria: (1) other comorbid mental disorders; (2) serious neurological or medical diseases, other sleep disorders; (3) Body Mass Index score > 30; (4) frequent jet lag; (5) contraindications for 3T MRI/TMS.

Behavioral assessments (e.g., PSQI, ISI, Beck Depression Inventory (BDI) and Beck Anxiety Inventory (BAI)) and all-night sleep PSG recordings of the patients were collected before and after active rTMS intervention. The HCs also underwent the same assessment.

### 2.2 PSG data acquisition and analysis

PSG was recorded on the Grael 4K system (Compumedics, Australia). The core montage included EEG (F3, F4, C3, C4, O1, O2, standard 10-20 system, referenced to bilateral linked mastoids), vertical and horizontal electrooculography (EOG), and submental electromyography (EMG). The sampling rate was 256Hz and impedances were kept below 10 kΩ.

The preprocessing was conducted, including removal of bad channels and down-sampling of the signal to 100Hz followed by filtering with a high- and low-pass filter of 0.4 and 35Hz respectively. Then the automatic sleep stage scoring was performed using YASA (18) and visually checked by a senior EEG expert according to the American Association for Sleep Research Manual of Interpretation of Sleep and Associated Events version 3 (AASM, https://aasm.org/). Several objective sleep measures were calculated according to the staging results, including sleep onset latency (SOL, sleep onset to first epoch of stage NREM in minutes), sleep efficiency (SE, total sleep time/time in bed), wake after sleep onset (WASO), the percentage of N2, N3, and REM sleep stages to total sleep time (N2%, N3% and REM%), arousal index (frequency of arousal per hour of sleep), awakenings (number of instances of two or more consecutive wake epochs after sleep onset).

### 2.3 Spectral analyses

The raw sleep EEG data were filtered with a band pass filter of 0–50Hz. Visually inspection was applied to exclude artifacts. The power spectral density was calculated by applying Welch method, with a 4-s Hanning window and 50% overlap, leading to a spectral resolution of 0.25Hz. Then we computed the absolute power by approximating the area under the PSD curve. Then the relative power was computed by dividing the absolute power of each frequency band, including delta (1–4Hz), theta (4–8Hz), alpha (8–12Hz), sigma (12–15Hz), beta (15–25Hz) and gamma (25-40Hz), by the sum of absolute power in all frequency bands. The relative power served as the main result of the current study because it is more sensitive and can reduce individual differences relative to absolute power (12).

Spectral analyses were performed on the 6 channels (F1, F2, C3, C4, O1, O2, referenced on bilateral linked mastoids), and the relative power of each frequency band in each sleep stage (N2, N3, and REM was calculated separately.

### 2.4 rTMS procedure

We used a MagPro R30 TMS stimulator with an ‘8’-shaped coil (MagVenture, Denmark), targeting the left DLPFC by the ‘5-cm rule’ to deliver pulses. The frequency of stimulations was set to 1Hz and the intensity was set at 80% of the resting motor threshold (motion threshold defined as the lowest stimulation intensity which can produce 5 finger movements out of 10 stimuli). A total of 150 strings of stimulation were administered, with each string consisting of 10 pulses, spaced 2 seconds apart, resulting in a total of 1500 pulses delivered over a 30-minute period. Twenty sessions of 1Hz rTMS on the left DLPFC were delivered over 4 consecutive weeks (one session per day, 5 days per week).

### 2.5 Statistical analysis

Statistical analyses were performed using SPSS version 24.0. At baseline, between-group differences in demographic and polysomnography variables, as well as sleep EEG relative power, were evaluated with unpaired, two-tailed t-test. Pearson correlation analysis was employed to examine the correlation between behavioral measures (PSQI, ISI, SOL, SE, BDI and BAI) and relative power of sleep EEG in patients with ID. The statistical analysis of differences in relative power and behavioral measures was conducted using paired t-tests to compare pre- and post-treatment data. Additionally, unpaired t-tests were employed to evaluate differences between the post-treatment ID patients and HCs. All values were represented as mean ± standard error of the mean. The level of significance was set at *P* < 0.05 (FDR corrected).

## 3 Results

### 3.1 Demographic and clinical characteristics

The detailed demographic and clinical characteristics of the participants were shown in **Table 1**. There were no significant differences in age, sex and education between HCs and ID patients. Before the rTMS treatment, the ID group had higher PSQI (t = 18.824, *P* < 0.001), ISI (t = 18.506, *P* < 0.001), BDI (t = 5.924, *P* < 0.001) and BAI (t = 5.116, *P* < 0.001) scores than HC, and also exhibited higher WASO (t = 2.722, *P* = 0.009), N2% (t = 2.961, *P* = 0.005) and awakenings (t = 2.033, *P* = 0.047), as well as a lower N3% (t = 2.601, *P* = 0.012). In addition, the difference of SE (t = 1.341, *P* = 0.186), SOL (t = 1.984, *P* = 0.053), REM% (t = 0.367, *P* = 0.715) and arousal index (t = 0.665, *P* = 0.509) was not significant (pre-rTMS vs. HC).

**Table 1:** Demographic information and behavioral measures of all participants.

	Patients with ID (n=26)		Healthy Controls (n=26)
	Pre-rTMS	Post-rTMS	
Age (years)	43.27 ± 2.09	–	40.92 ± 1.23
Gender (M/F)	11\15	–	11\15
Education (years)	12.90 ± 0.76	–	13.23 ± 0.49
PSQI	12.85 ± 0.45	7.96 ± 0.45***	2.88 ± 0.28***
ISI	16.31 ± 0.75	9.65 ± 0.70***	1.62 ± 0.25***
BDI	11.60 ± 1.24	8.6 ± 1.1**	3.37 ± 0.62***
BAI	34.97 ± 1.63	32.06 ± 1.12*	26.45 ± 0.35***
SE (%)	68.65 ± 3.00	73.25 ± 2.34*	73.59 ± 2.13
SOL (minutes)	26.02 ± 3.42	17.17 ± 2.93**	17.63 ± 2.48
WASO (minutes)	81.52 ± 9.60	87.17 ± 10.93	50.77 ± 5.95**
N2%	59.34 ± 1.94	58.37 ± 2.14	52.17 ± 1.45**
N3%	14.01 ± 1.56	14.61 ± 1.74	19.50 ± 1.43*
REM%	18.75 ± 1.60	16.59 ± 1.63	19.56 ± 1.49
Arousals Index	7.56 ± 0.50	7.40 ± 0.45	7.15 ± 0.39
Awakenings	21.38 ± 1.98	20.23 ± 1.64	16.35 ± 1.49*

Data are mean ± standard error.

^*^P<0.05, ^**^P<0.01, ^***^P<0.001.

Both asterisks in the post-rTMS column and the healthy controls column indicate a significant difference compared to the measurements of patients with ID before rTMS treatment.

ID, insomnia disorder; rTMS, repetitive transcranial magnetic stimulation; PSQI, Pittsburgh Sleep Quality Index; ISI, Insomnia Severity Index; BDI, Beck Depression Inventory; BAI, Beck Anxiety Inventory; SE, sleep efficiency; SOL, sleep onset latency; WASO, wake after sleep onset; N2%, the percentage of non-rapid eye movement sleep stage 2 sleep time to total sleep time; N3%, the percentage of non-rapid eye movement sleep stage 3 sleep time to total sleep time; REM%, the percentage of rapid eye movement sleep time to total sleep time.

After the rTMS treatment, ID group had significant improvements in both subjective sleep quality (PSQI, t = 7.305, *P* < 0.001; ISI, t = 10.605, *P* < 0.001) and objective sleep quality (SE, t =-2.640, *P* = 0.014; SOL, t = 2.883, *P* = 0.008). Significant reductions in BAI (t = 2.735, *P* = 0.011) and BDI (t = 3.161, *P* = 0.004) were also observed (pre-rTMS vs. post-rTMS). Compared to the HCs, there were no significant differences in SE (t = 0.105, *P* = 0.917), SOL (t = 0.120, *P* = 0.905), REM% (t = 1.325, *P* = 0.192), arousal index (t = 0.423, *P* = 0.674), and awakenings (t = 1.751, *P* = 0.086), whereas significant differences remained in PSQI (t = 9.605, *P* < 0.001), ISI (t = 10.770, *P* < 0.001), BAI (t = 4.780, *P* < 0.001), BDI (t = 4.220, *P* < 0.001), WASO (t = 2.924, *P* = 0.005), N2% (t = 2.394, *P* = 0.020) and N3% (t = 2.172, *P* = 0.035; post-rTMS vs. HC)).

### 3.2 Correlations between behavioral measures and sleep EEG relative power

**Figure 2:**
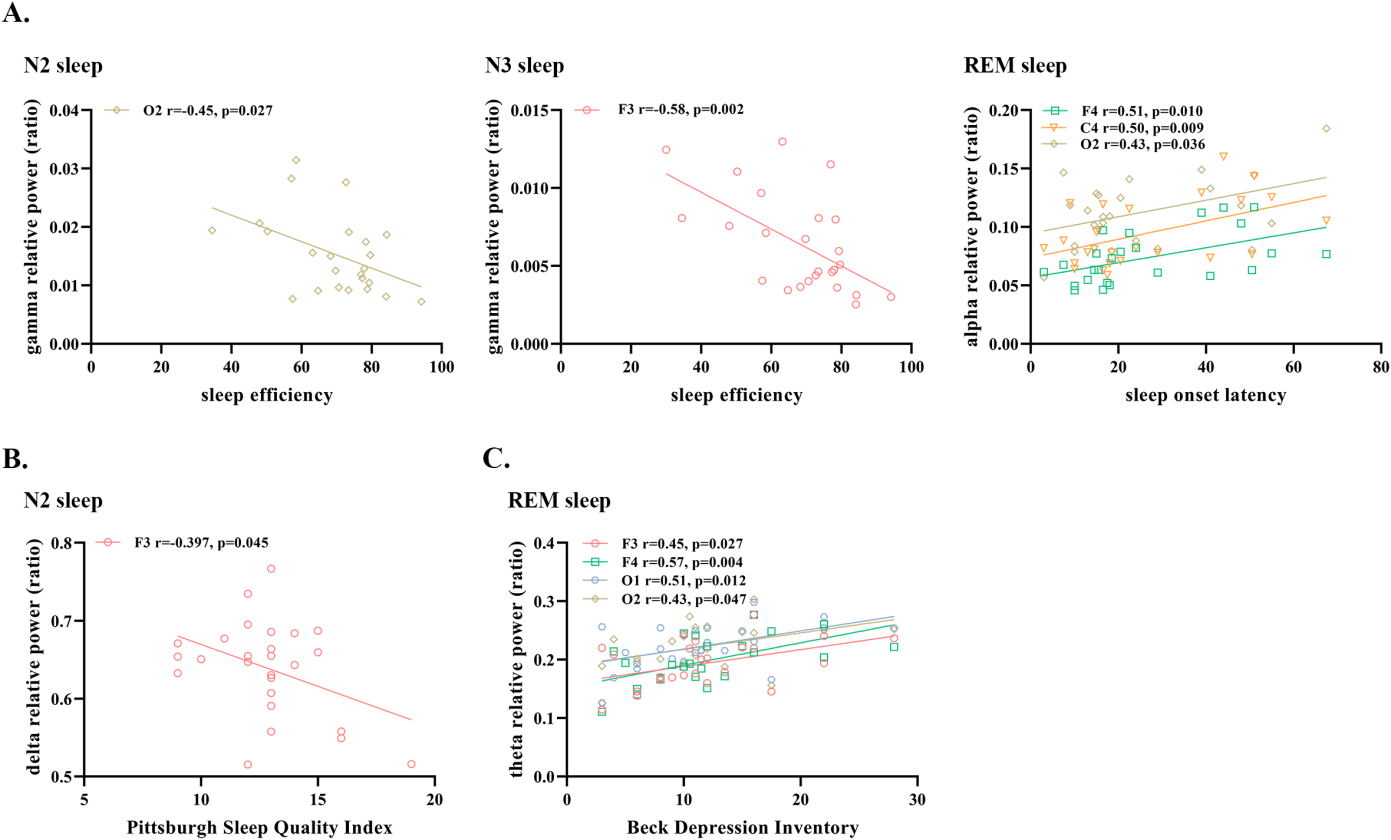
Significant correlations between sleep EEG relative power and **(A)** objective sleep measures **(B)** subjective sleep measures and **(C)** measures of depression and anxiety in patients with insomnia disorder. N2, Non-rapid Eye Movement Sleep Stage 2; N3, Non-Rapid Eye Movement Sleep Stage 3; REM, Rapid Eye Movement Sleep; EEG, electroencephalogram.

In terms of objective quality of sleep at baseline, negative correlations were observed in ID patients between SE and the relative power in the gamma band during N2 and N3 sleep. Our results also revealed that the alpha relative power of ID patients during REM sleep was positively correlated with SOL (**Figure 2A**).

In addition, we found a negative correlation between PSQI and delta relative power during N2 sleep, and a positive correlation between BDI and theta relative power during REM sleep (**Figures 2B, C**). All the above results were obtained after excluding the outliers.

### 3.3 Abnormities of sleep EEG relative power in patients with ID

Extensive differences in sleep EEG relative power between ID patients and HCs were observed, spanning N2, N3 and REM sleep stages and covering the frontal (F3, F4), central (C3, C4) and occipital (O1, O2) regions. Specifically, compared to HCs, significant excessive relative power in beta and gamma bands was observed at all six electrodes across the three distinct sleep stages in ID patients (*P* < 0.05, FDR corrected), except for relative power in the beta band at F4 in REM sleep (*P* = 0.085, FDR corrected). In addition, patients had lower relative power in the delta band during REM and N3 sleep (*P* < 0.05, FDR corrected, except for relative power at O2 during REM). Also, the relative power in the delta band at F4 in ID patients was significantly lower than HCs (*P* = 0.03, FDR corrected). Besides, our results revealed excessive relative power in the sigma band in N3 sleep at frontal and central areas (*P* < 0.05, FDR corrected). Detailed results were shown in **Table 2** and **Supplementary Table S1**.

**Table 2:** The difference of relative power between HCs (n=26) and patients with ID (n=26) across different frequency range and electrode sites.

Sleep Stage	Frequency /Electrode	F3	F4	C3	C4	O1	O2
REM	Delta	0.011^a*^	0.015^a*^	0.012^a*^	0.006^a**^	0.008^a**^	0.155
	Theta	0.833	0.482	0.383	0.314	0.100	0.843
	Alpha	0.155	0.344	0.152	0.067	0.176	0.295
	Sigma	0.220	0.514	0.137	0.062	0.594	0.365
	Beta	0.023^b*^	0.085	0.038^b*^	0.023^b*^	0.036^b*^	0.023^b*^
	Gamma	0.011^b*^	0.012^b*^	0.021^b*^	0.008^b**^	0.023^b*^	0.011^b*^
N2	Delta	0.066	0.030^a*^	0.169	0.061	0.137	0.097
	Theta	0.309	0.358	0.685	0.385	0.095	0.076
	Alpha	0.383	0.505	0.843	0.344	0.685	0.596
	Sigma	0.165	0.078	0.211	0.138	0.612	0.617
	Beta	0.012^b*^	0.035^b*^	0.012^b*^	0.021^b*^	0.026^b*^	0.038^b*^
	Gamma	0.011^b*^	0.023^b*^	0.004^b**^	0.008^b**^	0.008^b**^	0.021^b*^
N3	Delta	0.045^a*^	0.012^a*^	0.047^a*^	0.030^a*^	0.034^a*^	0.040^a*^
	Theta	0.125	0.062	0.100	0.100	0.071	0.093
	Alpha	0.121	0.105	0.118	0.058	0.088	0.097
	Sigma	0.036^b*^	0.011^b*^	0.038^b*^	0.035^b*^	0.062	0.088
	Beta	0.011^b*^	0.004^b**^	0.012^b*^	0.008^b**^	0.023^b*^	0.015^b*^
	Gamma	0.006^b**^	0.008^b**^	0.004^b**^	0.008^b**^	0.008^b**^	0.011^b*^

Data are p-value (FDR corrected).

a HC group is significantly higher than ID group.

b ID group is significantly higher than HC group.

^*^P<0.05, ^**^P<0.01.

HC, healthy control; ID, insomnia disorder; REM, Rapid Eye Movement Sleep; N2, Non-rapid Eye Movement Sleep Stage 2; N3, Non-rapid Eye Movement Sleep Stage 3.

### 3.4 rTMS treatment effects on sleep EEG relative power

**Figure 3:**
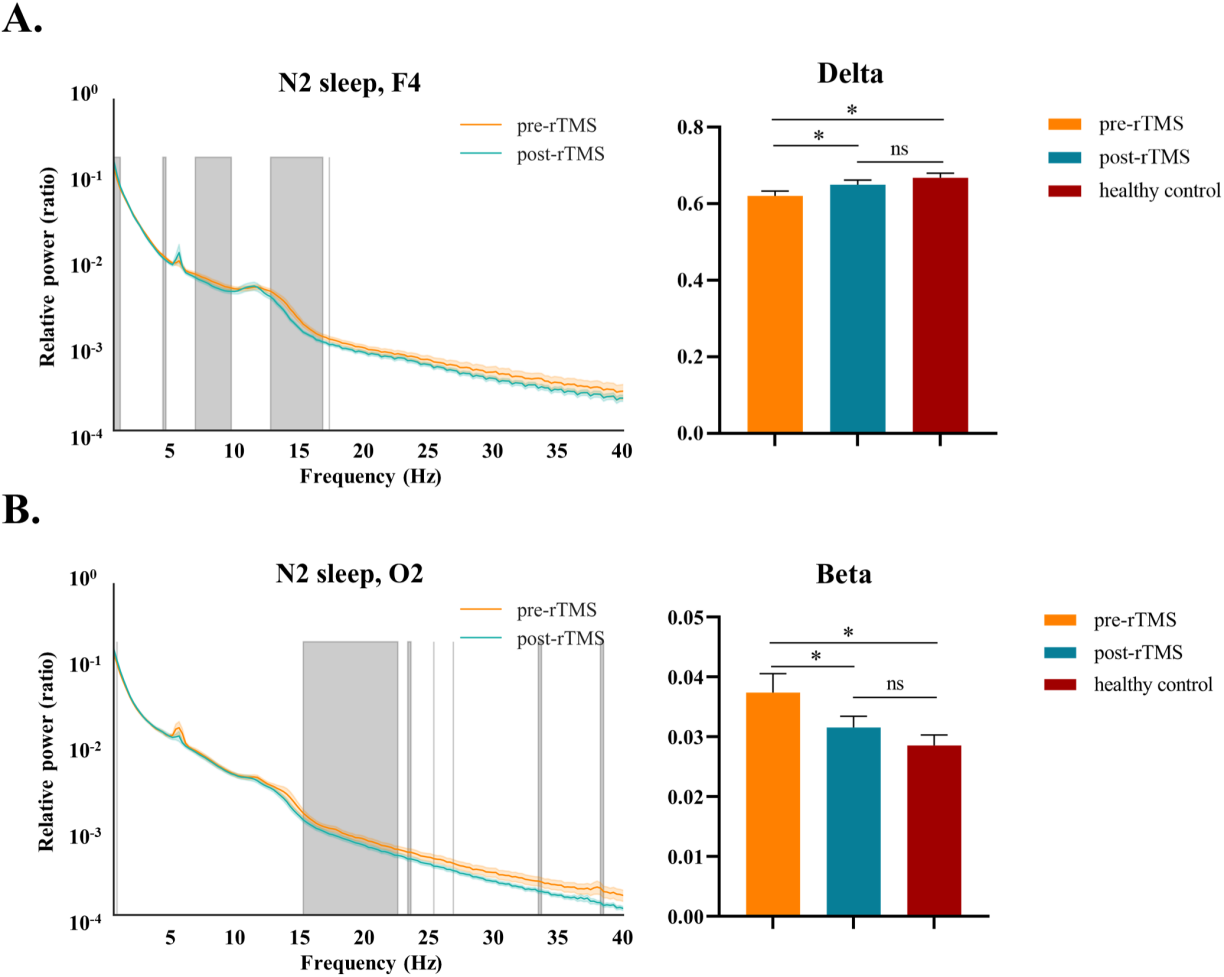
Comparison of sleep EEG relative power at **(A)** F4 and **(B)** O2 during N2 sleep in patients: Pre- vs. Post-rTMS treatment (not corrected) and healthy controls (FDR corrected). Curve graphs showed the average relative power of the patients in the artifact free epochs before and after treatment. Shaded area represented standard error of the mean, and grey overlays indicated significant differences. Histograms showed the frequency band with significant improvements in relative power. ns, not significant, *P < 0.05. rTMS, repetitive transcranial magnetic stimulation; N2, Non-rapid Eye Movement Sleep Stage 2; REM, Rapid Eye Movement Sleep.

After 1Hz rTMS treatment, significant increased relative power in the delta band (t = 2.362, *P* = 0.026) and reduced relative power in the beta band (t = 2.529, *P* = 0.018) during N2 sleep were observed (**Figure 3**). However, it did not pass the FDR correction. In addition, we did not observe significant changes in the high-frequency gamma band; rather, only a small portion of the high-frequency components at O2 during N2 sleep showed alterations (**Figure 3**).

## 4 Discussion

Since the widespread acceptance and high feasibility of EEG, coupled with the fact that abnormal EEG power spectra constitutes important objective pathological manifestations in individuals with insomnia, exploring the neurophysiological mechanisms through scalp EEG and investigating strategies to reverse these electrophysiological abnormalities may be crucial for understanding and treating ID. The current study examined the relative power of sleep EEG across six frequency bands during different sleep stages and investigated the effects of rTMS treatment on sleep EEG in patients with ID.

Elevated beta power in patients with ID during sleep has been widely reported (9, 19, 20), and is a reliable and objective biomarker of cortical hyperarousal (12). Consistently, in the current study, we observed that beta relative power was significantly higher in patients with ID during N2, N3 and REM sleep than in HCs, and was widely distributed in the frontal, central and occipital regions. Moreover, our results revealed that relative power of the Gamma band during NREM sleep was significant negatively correlated with SE, but not with subjective sleep quality and SOL. This suggests that excessive high-frequency relative power may be associated with the increased frequency of sleep stage shifts and increased brief waking periods and microarousals in patients with ID (21, 22). Besides, we observed excessive sigma oscillations during N3 sleep, although we did not find any correlation with behavioral measurements. Such abnormalities can be used as an objective marker of ID and might be a compensatory manifestation of maintaining the stability of N3 sleep (23). Slow-wave, including slow oscillations (<1Hz) and delta waves (1-4Hz), is a marker of diminishing arousal and a signature of NREM (13). Patients with ID may fail to perfectly reduce arousal in the poor sleep, as observed in the current study, the relative power of delta band was generally significant reduced over a broad spatial (frontal, central and occipital regions) distributions in REM and N3 sleep in ID patients. According to the above, our results revealed that EEG relative power abnormalities during NREM sleep in ID patients had different patterns in the N2 and N3 sleep stages, reflected in the delta and sigma bands.

Sleep problems are found to be associated with depression (24, 25). Transcriptomic profiles related to REM sleep and theta oscillations are found to overlap with predictors for depression (26, 27). Our results found significant correlations between relative power in the theta band during REM sleep and BDI scores. However, we did not observe significant difference in REM sleep theta relative power between patients with ID and HC, which may be due to the fact that the patients we recruited did not have comorbidity of depression, but only some had mild symptoms.

Behavioral efficacy of low frequency rTMS in insomnia disorder has been reported (17, 28). Consistently, a notable enhancement in both subjective and objective sleep quality was observed in the current study. Moreover, rTMS can also regulated brain function and neural oscillations (17, 29, 30). However, the effect of low-frequency rTMS on the relative power of EEG in sleeping brain remains unclear. Compared to the differences in relative power in the delta, beta and gamma bands that were widespread at baseline across various sleep stages and electrodes, the current study found that rTMS had a limited range of effects. Specifically, the restorations in the relative power of sleep EEG was observed exclusively in N2 sleep, suggesting that the N3 sleep, associated with deep sleep, exhibited lower susceptibility to rTMS. The stage N3 sleep was disrupted in prolonged episodes of insomnia, potentially requiring an extended course of treatment or rehabilitation for restoration. Furthermore, alterations in the relative power of the gamma band were not observed following rTMS treatment, possibly attributable to the limited influence of low-frequency rTMS, which may not extend to the broader spectrum of high-frequency gamma.

The modulation of rTMS to the brain could have spreading effects guided by the target region’s connectivity profile. As it was reported that TMS of prefrontal regions had an impact not only to the functional connectivity of the stimulated target but also on the non-stimulated occipital regions indirectly (31). In the current study, we found that after rTMS treatment, the relative power of sleep EEG abnormalities in the right frontal and occipital regions (F4, O2) were improved in patients with ID (uncorrected). However, no significant changes at the F3 electrode closest to the target of stimulation were observed. Prior researches have indicated a potential heightened vulnerability of the right hemisphere to disruptions related to insomnia (32–34), but further investigations are required to determine whether the right hemisphere is more susceptible to the effects of rTMS in patients with ID. It must be noted that these results should be interpreted with caution as they did not pass the FDR correction.

There are several limitations in our study. Firstly, the limited number of electrodes recording sleep EEG restricts the amount of information obtained from the brain. Employing a greater number of electrodes in future studies can facilitate more detailed and comprehensive investigations. Secondly, the current observational study did not include a sham group or a control group receiving rTMS intervention. Thirdly, PSG was performed only once before and after treatment, which may have made the results less reliable in patients with high night-to-night variability. Additional PSG or sleep diaries should be considered in future studies. Finally, the current study does not allow us to discern whether the improvement in sleep quality occurred prior or subsequent to the changes in cortical power patterns or simultaneously. Future studies should enhance dynamic monitoring of patients during rTMS treatment and investigate the direct effects of rTMS on patients' EEG using techniques such as TMS-EEG.

In conclusion, our study provides further evidence of the complex neurophysiological abnormalities in patients with ID, highlighting distinct alterations in sleep EEG relative power across different sleep stages and its correlations with baseline behavioral measures. While the findings underscore the potential of rTMS in improving certain aspects of sleep EEG, particularly during N2 sleep, the limited effects observed in deeper sleep stages and high-frequency oscillations suggest that rTMS might require a more targeted or prolonged intervention to achieve broader neurophysiological benefits.

## Data Availability

The data, such as PSG recordings, reported in this study is available from the corresponding authors on reasonable request. Analyses were conducted mainly by using YASA toolbox (Python); code required to reanalyze the data reported in this article is available from corresponding authors on request. Requests to access the datasets should be directed to XS, sxn108@163.com.
