# Supplementary material for: Restoration of abnormal sleep EEG power in patients with insomnia disorder after 1Hz rTMS over left DLPFC
Source: Front Psychiatry. 2024 Sep 10;15:1431837. doi: 10.3389/fpsyt.2024.1431837 (PMC11419987; doi:10.3389/fpsyt.2024.1431837)
Supplement: Supplementary file 1 [file Table1.docx]

**Supplemental Tables**

**Table S1.** The relative power of HCs (n=26) and patients with ID (n=26) across different frequency range and electrode sites.

| Sleep Stage | Frequency /Electrode | F3 | F4 | C3 | C4 | O1 | O2 |
| --- | --- | --- | --- | --- | --- | --- | --- |
| Relative power of HCs | | | | | | | |
| REM | Delta | 0.611 (0.056) | 0.602 (0.047) | 0.587 (0.056) | 0.589 (0.057) | 0.572 (0.050) | 0.546 (0.123) |
|  | Theta | 0.191 (0.022) | 0.190 (0.019) | 0.207 (0.021) | 0.202 (0.021) | 0.208 (0.021) | 0.237 (0.154) |
|  | Alpha | 0.066 (0.014) | 0.068 (0.016) | 0.080 (0.022) | 0.079 (0.021) | 0.101 (0.025) | 0.102 (0.032) |
|  | Sigma | 0.027 (0.006) | 0.028 (0.005) | 0.030 (0.007) | 0.030 (0.007) | 0.035 (0.007) | 0.034 (0.009) |
|  | Beta | 0.064 (0.021) | 0.069 (0.017) | 0.060 (0.016) | 0.063 (0.015) | 0.056 (0.012) | 0.053 (0.015) |
|  | Gamma | 0.041 (0.014) | 0.044 (0.014) | 0.036 (0.011) | 0.037 (0.011) | 0.029 (0.008) | 0.027 (0.006) |
| N2 | Delta | 0.678 (0.070) | 0.668 (0.062) | 0.659 (0.074) | 0.659 (0.065) | 0.639 (0.073) | 0.635 (0.077) |
|  | Theta | 0.158 (0.025) | 0.160 (0.023) | 0.172 (0.035) | 0.171 (0.029) | 0.190 (0.038) | 0.193 (0.041) |
|  | Alpha | 0.079 (0.026) | 0.084 (0.025) | 0.086 (0.026) | 0.087 (0.025) | 0.094 (0.027) | 0.094 (0.027) |
|  | Sigma | 0.038 (0.018) | 0.039 (0.016) | 0.041 (0.016) | 0.041 (0.015) | 0.039 (0.015) | 0.038 (0.015) |
|  | Beta | 0.031 (0.011) | 0.034 (0.012) | 0.030 (0.010) | 0.031 (0.009) | 0.028 (0.008) | 0.029 (0.009) |
|  | Gamma | 0.015 (0.005) | 0.015 (0.005) | 0.012 (0.003) | 0.012 (0.004) | 0.010 (0.003) | 0.011 (0.004) |
| N3 | Delta | 0.844 (0.058) | 0.840 (0.052) | 0.850 (0.047) | 0.849 (0.046) | 0.834 (0.051) | 0.833 (0.049) |
|  | Theta | 0.098 (0.025) | 0.100 (0.019) | 0.098 (0.024) | 0.098 (0.022) | 0.111 (0.030) | 0.113 (0.029) |
|  | Alpha | 0.034 (0.023) | 0.037 (0.024) | 0.031 (0.016) | 0.031 (0.016) | 0.034 (0.015) | 0.034 (0.014) |
|  | Sigma | 0.012 (0.008) | 0.013 (0.008) | 0.012 (0.006) | 0.012 (0.006) | 0.011 (0.006) | 0.011 (0.006) |
|  | Beta | 0.008 (0.004) | 0.008 (0.004) | 0.007 (0.003) | 0.007 (0.003) | 0.007 (0.003) | 0.007 (0.003) |
|  | Gamma | 0.004 (0.002) | 0.004 (0.002) | 0.003 (0.001) | 0.003 (0.001) | 0.003 (0.001) | 0.003 (0.001) |
| Relative power of IDs | | | | | | | |
| REM | Delta | 0.545 (0.088) | 0.540 (0.090) | 0.504 (0.118) | 0.499 (0.102) | 0.508 (0.077) | 0.502 (0.087) |
|  | Theta | 0.193 (0.039) | 0.197 (0.040) | 0.227 (0.100) | 0.216 (0.058) | 0.224 (0.038) | 0.232 (0.055) |
|  | Alpha | 0.074 (0.021) | 0.074 (0.022) | 0.092 (0.031) | 0.094 (0.027) | 0.114 (0.033) | 0.112 (0.030) |
|  | Sigma | 0.029 (0.007) | 0.029 (0.007) | 0.035 (0.013) | 0.036 (0.012) | 0.036 (0.007) | 0.036 (0.008) |
|  | Beta | 0.086 (0.035) | 0.085 (0.036) | 0.081 (0.038) | 0.087 (0.041) | 0.071 (0.027) | 0.072 (0.031) |
|  | Gamma | 0.073 (0.045) | 0.076 (0.049) | 0.061 (0.042) | 0.068 (0.044) | 0.048 (0.034) | 0.046 (0.030) |
| N2 | Delta | 0.639 (0.062) | 0.620 (0.067) | 0.627 (0.074) | 0.615 (0.075) | 0.608 (0.061) | 0.593 (0.081) |
|  | Theta | 0.166 (0.028) | 0.167 (0.031) | 0.176 (0.038) | 0.179 (0.033) | 0.209 (0.035) | 0.220 (0.053) |
|  | Alpha | 0.087 (0.029) | 0.090 (0.026) | 0.088 (0.026) | 0.095 (0.027) | 0.091 (0.024) | 0.090 (0.023) |
|  | Sigma | 0.046 (0.018) | 0.050 (0.020) | 0.048 (0.022) | 0.050 (0.022) | 0.042 (0.020) | 0.041 (0.020) |
|  | Beta | 0.040 (0.010) | 0.045 (0.018) | 0.041 (0.015) | 0.041 (0.017) | 0.036 (0.011) | 0.037 (0.016) |
|  | Gamma | 0.022 (0.009) | 0.028 (0.023) | 0.019 (0.008) | 0.020 (0.010) | 0.015 (0.006) | 0.018 (0.012) |
| N3 | Delta | 0.806 (0.057) | 0.792 (0.057) | 0.811 (0.068) | 0.809 (0.062) | 0.791 (0.068) | 0.793 (0.067) |
|  | Theta | 0.111 (0.028) | 0.114 (0.028) | 0.113 (0.033) | 0.112 (0.030) | 0.130 (0.035) | 0.131 (0.038) |
|  | Alpha | 0.045 (0.021) | 0.049 (0.022) | 0.041 (0.024) | 0.043 (0.024) | 0.046 (0.027) | 0.045 (0.026) |
|  | Sigma | 0.019 (0.010) | 0.021 (0.010) | 0.018 (0.011) | 0.018 (0.011) | 0.017 (0.010) | 0.015 (0.009) |
|  | Beta | 0.012 (0.006) | 0.014 (0.007) | 0.011 (0.007) | 0.011 (0.006) | 0.011 (0.008) | 0.010 (0.005) |
|  | Gamma | 0.007 (0.004) | 0.009 (0.009) | 0.005 (0.003) | 0.006 (0.003) | 0.005 (0.003) | 0.005 (0.004) |

Data are mean (standard deviation).

*Abbreviations*: HC, healthy control; ID, insomnia disorder; REM, Rapid Eye Movement Sleep; N2, Non-rapid Eye Movement Sleep Stage 2; N3, Non-rapid Eye Movement Sleep Stage 3.
